# The Multifaceted Role of Baicalein in Cancer Management through Modulation of Cell Signalling Pathways

**DOI:** 10.3390/molecules27228023

**Published:** 2022-11-18

**Authors:** Arshad Husain Rahmani, Ahmad Almatroudi, Amjad Ali Khan, Ali Yousif Babiker, Malak Alanezi, Khaled S. Allemailem

**Affiliations:** 1 Department of Medical Laboratories, College of Applied Medical Sciences, Qassim University, Buraydah 51542, Saudi Arabia; 2 Department of Basic Health Sciences, College of Applied Medical Sciences, Qassim University, Buraydah 51542, Saudi Arabia; 3 Department of Dentistry, Dr. Sulaiman Al Habib Medical Group, Qassim 51431, Saudi Arabia

**Keywords:** baicalein, cancer, cell signaling pathways, synergistic effects, bioavailability

## Abstract

The roles of medicinal plants or their purified bioactive compounds have attracted attention in the field of health sciences due to their low toxicity and minimal side effects. Baicalein is an active polyphenolic compound, isolated from *Scutellaria baicalensis,* and plays a significant role in the management of different diseases. Epidemiologic studies have proven that there is an inverse association between baicalein consumption and disease severity. Baicalein is known to display anticancer activity through the inhibition of inflammation and cell proliferation. Additionally, the anticancer potential of baicalein is chiefly mediated through the modulation of various cell-signaling pathways, such as the induction of apoptosis, autophagy, cell cycle arrest, inhibition of angiogenesis, signal transducer and activator of transcription 3, and PI3K/Akt pathways, as well as the regulation of other molecular targets. Therefore, the current review aimed to explore the role of baicalein in different types of cancer along with mechanisms of action. Besides this, the synergistic effects with other anti-cancerous drugs and the nano-formulation based delivery of baicalein have also been discussed.

## 1. Introduction

**Figure 1:**
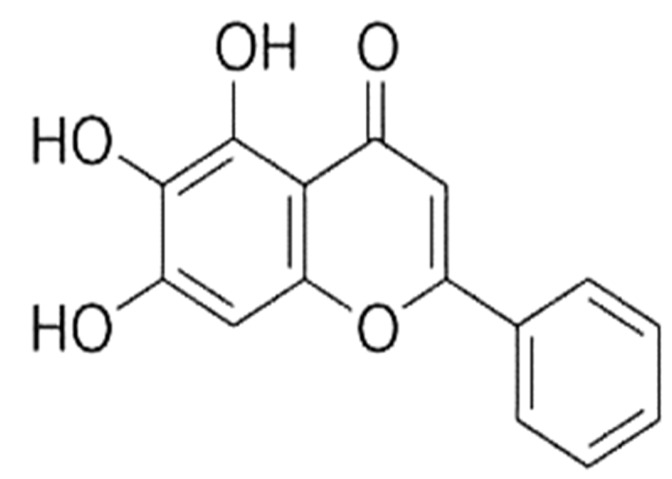
Chemical structure of baicalein.

Cancer is a significant health problem globally; it refers to a group of diseases triggered by abnormal cell growth with invasive potentials [1]. In addition to causing serious adverse effects to the patient’s body, cancer also places a massive economic burden on patients, and the burden on developing countries is rising [2]. Several endogenous factors such as hormones, genetic predisposition, inherited mutations, immune conditions, etc., and exogenous factors such as nutrition, environment, tobacco, lifestyle, diet, obesity, sun exposure, chemicals, radiation, as well as infectious organisms, play significant roles in the development of cancer [3,4,5]. Herbs or bioactive ingredients of medicinal plants play a role in disease management with fewer side effects. Previous findings suggest that the natural compounds or chief ingredients of medicinal plants play roles in health management through the modulation of oxidative stress, inflammation [6,7,8] and cell signaling pathways [9,10,11]. Moreover, as per a wide range of experiments, phytochemicals such as polyphenols, flavones, and flavonoids hold substantial anti-cancer potential, which can be used against various cancers [12]. The currently applied mode of treatment in cancer includes anti-cancerous drugs, targeted therapy, hormone therapy, and radiation therapy, which are effective in cancer treatment but also cause various adverse complications in the body. In this regard, the use of medicinal plants or their bioactive compounds based on in vitro and clinical studies can have anti-cancerous effects through targeting several signaling molecules. Baicalein (5,6,7-trihydroxyflavone), CAS No. (491-67-8) (Figure 1), is one of the active flavonoids principally found in dried roots of the traditional herb *Scutellaria baicalensis* [13], and it has attracted considerable attention for its reported ability to suppress cellular proliferation and induce apoptosis [14,15,16]. Moreover, baicalein plays an anti-cancerous role through modulating various cell signaling pathways, such as p-mTOR, p-Akt, p-IκB, and NF-κB, the protein expressions of which were significantly downregulated [17]. Baicalein induced AIF as well as Endo G release from mitochondria demonstrating that baicalein stimulates apoptosis via the caspase-independent pathway, whereas undergoing apoptosis, there was a noteworthy accumulation of G2/M cells [18] and decreased angiogenic factors such as vascular endothelial growth factor expression in cancer cell [19]. This review describes the molecular mechanisms of baicalein in different types of cancer through modulating various cell signaling pathways.

## 2. Mechanism of Action of Baicalein in Cancer Prevention and Treatment

The anti-cancerous role of baicalein operates through modulating various cell signaling pathways, as described below.

### 2.1. Inflammation

The abnormal regulation of molecular pathways involved in inflammation shows a vital association with cancer development and progression [20]. Interleukin-1β, Interlikin-6, Tumor necrosis factor-alpha (TNF-α), growth factors, chemokines and reactive oxygen species (ROS) play a significant role in the proliferation, metastasis, as well as chemo resistance of cancer cells through the stimulation of transcription factors such as mitogen-activated protein kinases (MAPKs), mTOR and NF-κB [21]. Moreover, the critical association between chronic inflammation and cancer is evidently explained by the fact that major pro-inflammatory transcription factors such as nuclear factor-κB, as well as signal transducers and activators of transcription 3, can be triggered by many of these vital cancer risk factors [22]. 

**Figure 2:**
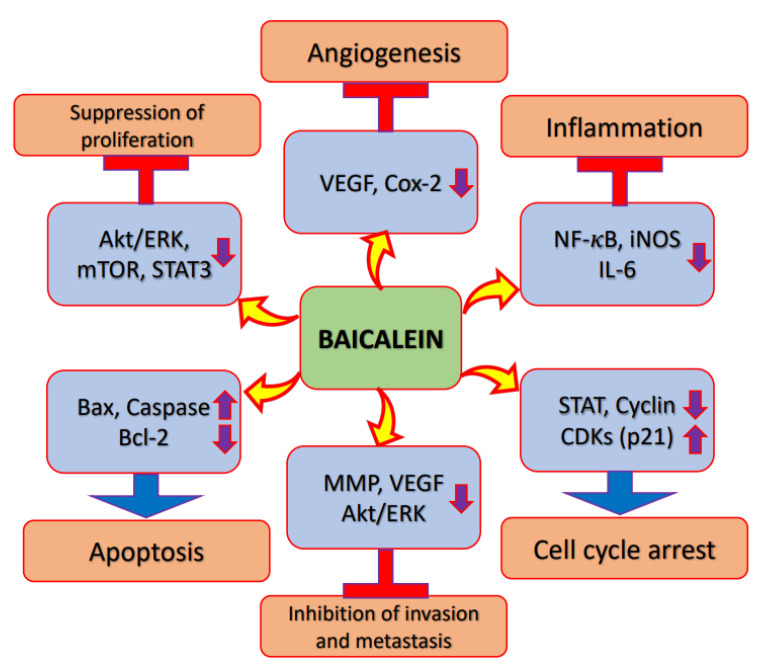
Anticancer effects of baicalein through the modulation of cell signaling pathways.

Cyclooxygenase-2 is an inducible enzyme and is linked with carcinogenesis and inflammatory diseases; it is supposed to promote angiogenesis and tissue tumor invasion [23,24]. However, the regulation of the inflammation process is a crucial step towards the inhibition of cancer development and growth. Baicalein has an important inhibitory effect on DMBA/TPA-mediated tumor promotion, and pretreatment with baicalein inhibits inflammatory cell production in DMBA/TPA-induced skin/tumors. Overall, it has been reported that baicalein inhibits skin tumorigenesis through suppressing inflammation and proliferation, and promoting apoptosis [25]. Furthermore, baicalein decreased the activation of nuclear factor-κB (*NF*-*κB*) via Peroxisome proliferator-activated receptor gamma (PPAR-γ) activation. These findings suggest that the anti-inflammatory effects of baicalein might be initiated via PPARγ activation. The administration of baicalein meaningfully decreases the incidence of tumor formation and baicalein is one of the important candidates in the prevention of inflammation-linked colon carcinogenesis [26]. The administration of baicalein meaningfully counteracted benzo[*a*]pyrene-induced increases in cytokines, such as iNOS, TNF-α, and interleukin-1β, in pulmonary carcinogenesis [27] (Figure 2 and Table 1).

### 2.2. Akt/PI3K/mTOR Signalling Pathway

Phosphatidylinositol-3-kinase (PI3K)/Akt/mammalian target of rapamycin (mTOR) signaling is one of the most important intracellular pathways, and it regulates cell growth, motility, survival, metabolism, and angiogenesis [28,29]. The activation of the PI3K/Akt/mTOR pathway contributes to the development of tumors and resistance to anti-cancer therapies [29]. Natural products or bioactive compounds play a significant role in cancer management through the inactivation of signaling pathways such as PI3K/Akt/mTOR. The proliferation of the gastric cancer cell line was inhibited by baicalein in a time- as well as dose-dependent fashion. Baicalein treatment pointedly decreased the phosphorylation of PI3K as well as Akt proteins. The study concluded that baicalein might inhibit the signaling pathway PI3K/Akt [30]. In order to clarify the causal molecular mechanism of baicalein-caused autophagy and apoptosis, the protein levels linked with the Akt signaling pathway PI3K were studied. The results suggest that p-mTOR, p-Akt, p-IκB, and NF-κB protein expressions were decreased as compared to the control group. In addition, the p-mTOR/mTOR and p-Akt/Akt ratios were also decreased in a dose- and time-dependent way. Together, these results demonstrate that baicalein controls the autophagy and apoptosis of cancer cells through the PI3K/Akt pathway [17] (Figure 2 and Table 1).

### 2.3. Apoptosis

Apoptosis is a programmed cell death response, and it plays a critical role in development as well as homeostasis [31]. Once the balance is altered, activated antiapoptotic signal pathway or pro-apoptosis pathway deficiency can cause uncontrolled proliferation of the cell, therapeutic resistance, and the recurrence of cancer cells [32]. 

Natural compounds play a vital role in the regulation of programmed cell death/apoptosis through the removal of tumor cells and the control the tumor growth. Caspase-initiated apoptosis has been noted as a mechanism of the inhibition of growth via baicalein. Baicalein-treated groups show the expression of cleaved caspase-3, the activity of which is even more significant with the addition of chloroquine. Over all, these findings suggest that the caspase-3 pathway of apoptosis with baicalein is improved with the inhibition of autophagy [33]. Moreover, it was reported that the quantity of apoptotic cells was meaningfully improved in the baicalin treatment group [34]. Baicalein decreased migration, invasion, cell viability and proliferation, and increased G1 phase numbers, apoptosis and the expression levels of p27, p21, cleaved caspase 3/9, Bax, ZO-1 and E-cad, in a dose-dependent way [35]. Baicalein showed a role in the activation of apoptosis via inducing intracellular reactive oxygen species generation, and the production of reactive oxygen species scavenger *N*-acetyl-cysteine, superoxide dismutase and glutathione, while apparently inhibiting intracellular ROS production, accordingly attenuating the baicalein-induced apoptosis. Moreover, baicalein induces mitochondrial fragmentation, which leads to cell apoptosis. Overall, the results suggest that baicalein treatment shows strong links with apoptosis and reactive oxygen species, which induce BNIP3 expression in cancer cells, and further suggest that baicalein has a powerful potential for use as an anti-osteosarcoma drug [36] (Figure 2 and Table 1).

### 2.4. Cell Cycle

Cell cycle checkpoints participate in monitoring as well as controlling the cell cycle’s progression [37,38,39,40,41]. Various natural products play a substantial part in cancer management through cell cycle arrest. Baicalein might inhibit cell proliferation in a time- and dose-dependent fashion. Moreover, it has been noted that baicalein induces G1 cell cycle arrest through decreasing cyclin-dependent kinase 4 and cyclin D1 [42]. The growth of lung squamous cell carcinoma was inhibited by baicalein in a dose-dependent way. Cell cycle analysis showed that treatment with 50 μM baicalein caused an increase in the S-phase cell population. During the S-phase arrest, analysis of cell cycle regulatory molecules established that baicalein reduced the levels of cdk 4, cyclin-D1 and -B1. Additionally, baicalein meaningfully induced cell apoptosis, and the results suggest that baicalein inhibits proliferation via apoptosis and S-phase arrest [43]. Different concentrations of baicalein were examined in cervix cancer, including SiHa and HeLa cells. The results showed the inhibition of cell transition from the G0/G1 phase to the S phase, suggesting that baicalein treatment caused the hindrance of SiHa and HeLa in the cell cycle. Overall, baicalein inhibited cell cycle progression and cell growth, and promoted apoptosis of cancer cells [44]. Another study reported that baicalein stimulated apoptosis in hepatoma cells as baicalein induced AIF as well as Endo G release from mitochondria demonstrating that baicalein stimulates apoptosis via the caspase-independent pathway, whereas undergoing apoptosis, there was a noteworthy accumulation of G2/M cells [18]. Moreover, baicalein controls cyclin D1 transcription through a β-catenin-dependent mechanism, leading to impaired cancer cell proliferation and cell cycle arrest at the G0/G1 phase [19] (Figure 2 and Table 1).

### 2.5. Angiogenesis

Angiogenesis Akt is an important factor of tumor growth and progression [45]. Among the pro-angiogenic factors, vascular endothelial growth factor (VEGF) is the most important mitogen for vascular endothelium [46], and the overexpression of VEGF has been noticed in tumors [47,48]. However, the regulation of angiogenesis is vital in the inhibition of tumor growth. Natural compounds have proven their anti-cancerous activities through anti-angiogenesis. Baicalin as well as baicalein meaningfully inhibit the vascular endothelial growth factor’s expression, and the inhibitory effect increases with increased concentrations. Then again, both compounds led to increased VEGF expression in normal cells. VEGF protein levels were inhibited to 46%, 8% and 7% in CP70 cells and 52%, 9% and 7% in OVCAR-3 cells by 40 μM, 80 μM and 160 μM baicalein treatments, respectively [49]. Baicalein treatment caused significant decreases in the density of blood vessels in the tumor models. Intratumoral blood vessel density was 37.56% in the untreated group, while it was 21.24% in the baicalein-treated group, and this observation was made in mice injected with B16F10 cells. The mice injected with LLC cells showed a pattern similar to the B16F10-injected group, with intratumoral blood vessel densities of 14.88% in the untreated group, and 9.51% in the baicalein-treated group. These findings suggest that baicalein treatment powerfully inhibited vasculature formation during tumor progression [50]. The histological analysis of tumor xenografts revealed decreased expressions of both vascular endothelial growth factor and 12-lipoxygenase proteins in baicalein-treated tumors. A significant decrease in both micro-vessel density and mitotic index was observed in the baicalein treatment group. Gene expression profiling demonstrated a decrease in FGFR2 as well as vascular endothelial growth factor following baicalein treatment [51] (Figure 2 and Table 1).

### 2.6. Autophagy

In cancer cells, autophagy suppresses tumorigenesis by inducing cell death and inhibiting cancer cell survival, and it also facilitates tumorigenesis via tumor growth and promoting cancer cell proliferation [52,53]. Autophagy in cancer cells is recognized as an important factor, but is a double-edged sword, since in the initial stages of tumorigenesis, it might act as a tumor-suppressor through breaking down actually harmful agents or damaged organelles, therefore preventing the spread of damage as well as DNA alterations [54]. Baicalein, a flavonoid and aglycon hydrolyzed from baicalin, causes human cancer cell death via the induction of autophagy—cell death induced by baicalein was completely reversed through a decrease in the expression levels of significant molecules in autophagy, including vacuolar protein sorting [34], Beclin1, autophagy-related (Atg)5, and Atg7. Moreover, baicalein induced autophagosome formation, and autophagic flux. These results offer a new mechanistic understanding of the anti-cancer functions of autophagy inducers, including baicalein, which may be used as therapeutics in cancer treatment [55]. The combination of cisplatin and baicalein decreased invasive potential and encouraged autophagy and apoptosis, and this effect was stronger than that of cisplatin or baicalein alone. Moreover, baicalein enhanced the cisplatin sensitivity of SGC-7901/DDP gastric cancer cells by inducing autophagy and apoptosis through the Akt/mTOR and Keap 1/Nrf2 pathways [56]. Baicalein induced cell apoptosis in a concentration-dependent fashion, and autophagic cell death occurred in cancer cells in the treatment with baicalein [57]. The role of baicalein as an anti-cancer agent was examined, focusing on thyroid cancer. It was demonstrated that baicalein upregulated the expressions of Atg5, Beclin-1, p62, Atg12, and caspase-3 and -8, and decreased the expression levels of Bcl-2/Bax, before inducing autophagy and apoptosis in the FRO cells via regulating the PI3K/Akt and ERK pathways [58].

### 2.7. Signal Transducer and Activator of Transcription 3 (*STAT3*)

Signal transducer and activator of transcription 3 (STAT3) is a transcription factor that controls cell growth and survival through controlling the expressions of target genes [59]. Additionally, STAT3 shows oncogene activity, which is constitutively active in cancers [60,61]. Baicalein inhibits the signal transducer and activator of transcription 3 transcriptional activity and its phosphorylation, and additionally shows anti-proliferative effects. Moreover, some studies suggest baicalein as a striking phytochemical compound for decreasing the metastatic potential of breast cancer cells through regulating signal transducer and activator of transcription 3 activity [62]. The potential effects and mechanisms of baicalein in controlling the radiosensitivity of cancer cells were examined. Baicalein treatment reduced the cell viability of 10-Gy X-ray-irradiated cervix cancer Hela cells, partly reducing the effect of miR-183, and it also prevented EMT in irradiated cells and increased apoptosis. The Tyrosine (Tyr) residue 705 of STAT3 and Y1007/8 in JAK2 were phosphorylated, leading to the high expression of JAK2/STAT3, which was decreased through irradiation as well as baicalein treatment. Baicalein played a role in the inhibition of cell viability and epithelial–mesenchymal transition, and it induced cell apoptosis in cancer cells, via upregulating miR-183 through the inactivation of the signaling pathway JAK2/STAT3 [63] (Figure 2 and Table 1).

### 2.8. Wnt/β-Catenin Pathway

Baicalein inhibits the proliferation of and increases in miR-25 expression, and regulates the Wnt/β-catenin pathway, in the osteosarcoma cell line. Moreover, baicalein and miR-25 decreased the expressions of Axin2 and β-catenin, whereas the expression of GSK-3β was increased. Additionally, the downregulation of miR-25 decreased the GSK-3β expression, whereas Axin2 and β-catenin expression increased [64]. Total β-catenin expression was suggestively reduced by baicalein in osteosarcoma cells. The translocation of β-catenin from the cytoplasm to the nucleus stimulates the signaling of Wnt, and it is reported that the expression of cytoplasmic β-catenin was unchanged by treatment with baicalein. Besides this, Oct3/4, CD44, CCND2, CCND1, and surviving are downstream target genes of Wnt/β-catenin, and their expressions were suppressed by baicalein administration. All these outcomes suggest that baicalein improved the canonical Wnt/β-catenin pathway through altering its translocation from cytoplasm to nucleus [65].

### 2.9. Tumor Suppressor Genes

Tumor suppressor genes play a significant role in the inhibition of cancer development and growth. Therefore, the loss of tumor suppression genes or their inactivation was noticed in many cancers [66,67]. However, tumor suppressor genes are vital in the control of cancer development. PTEN is encoded on chromosome 10q23, a region where the loss of heterozygosity frequently occurs in cancer [68]. Baicalein increased the sensitivity of gastric cancer cells to 5-fluorouracil treatment under hypoxia conditions. Additionally, hypoxia-induced Akt phosphorylation was inhibited by baicalein through enhancing PTEN accumulation, and in this way reducing hypoxia-inducible factor-1α expression in cancer cells. These findings together suggest that the inhibition of glycolysis through the regulation of the PTEN/Akt/HIF-1α signaling pathway might be one of the possible mechanisms whereby baicalein reverses 5-fluorouracil resistance in cancer cells under hypoxia [69]. A lung cancer-based study showed that baicalein upregulated PTEN expression, downregulated miR-424-3p, and downregulated the expressions of PI3K and p-Akt in H460 and A549 cells. A dual-luciferase reporter assay established that PTEN is a target gene of miR-424-3p, and the overexpression of miR-424-3p or the silencing of PTEN partly reduce the effects of baicalein on cancer cells [70]. 

### 2.10. ERK/p38/MAPK Pathway

The mitogen-activated protein kinase (MAPK) pathway is an intracellular signal transduction pathway that controls cellular processes, including cell growth, cell proliferation, cell migration, stress response and apoptosis, in response to numerous extracellular stimuli [71,72,73]. It consists of pathways involving extracellular-signal-regulated kinase 1 and 2, and the p38 MAPK signaling pathway [74]. The p38 MAPK pathways are activated in response to several environmental and cellular stresses, other signals, and inflammation [75,76]. To examine whether the effects of the mixture of baicalin and baicalein treatment include the stimulation of ERK or p38 MAPK, we studied the phosphorylation of p38 MAPK as well as ERK. The expressions of p-p38 and p-ERK were boosted by around 2.85- and 2.96-fold, respectively, by the combined drug treatment in comparison to the control. These findings suggest that the combined drug treatment affects p-ERK and p-p38 [77]. In hepatocellular carcinoma cells, we explored the effects of baicalein on the ERK pathway. The Western blotting results reveal that baicalein reduced the phosphorylation of ERK1/2 and MEK1 in a concentration-dependent fashion [78].

**Table 1:** Key mechanism of baicalein in tumor management through the enhancement of cell signaling pathways.

Genes/Pathway	Mechanism	Refs.
**NF-Κb**	Baicalein decreased the activation of NF-κB and the anti-inflammatory effects of baicalein might be initiated via PPARγ activation.	[26]
**Matrix metalloproteinase**	Baicalein treatment efficiently denies B(a)P-induced upregulated expression of matrix metalloproteinase-2 and 9.	[27]
**PI3K/Akt**	Baicalein treatment notably decreased the phosphorylation of PI3K and Akt proteins. The study concluded that baicalein might inhibit the PI3K/Akt signaling pathway.	[30]
	Baicalein regulates the autophagy and apoptosis of cancer cells through the PI3K/Akt pathway.	[17]
**Apoptosis**	In baicalein-treated groups, the expression of cleaved caspase-3 and caspase-3 activity was even more significant with chloroquine addition. Over all, baicalein treatment improved the caspase-3 pathway of apoptosis through autophagy inhibition.	[33]
	Baicalein decreased migration, invasion, cell viability and proliferation, and increased G1 phase numbers, apoptosis and the expression levels of p27, p21, cleaved caspase 3/9, Bax, ZO-1 and E-cad, in a dose-dependent way.	[35]
	Apoptosis and reactive oxygen species caused BNIP3 expression in cancer cells with baicalein treatment. The findings advocate that baicalein has powerful potential as an anti-osteosarcoma drug.	[36]
	Baicalein induced AIF as well as Endo G release from mitochondria demonstrating that baicalein stimulates apoptosis via the caspase-independent pathway, whereas undergoing apoptosis, there was a noteworthy accumulation of G2/M cells.	[18]
**Cyclin-dependent kinase**	Baicalein induces G1 cell cycle arrest through decreasing cyclin-dependent kinase 4 and cyclin D1.	[42]
	Cell cycle-regulatory molecule studies established that baicalein decreased the levels of cdk 4, cyclin D1 and B1.	[36]
**VEGF**	VEGF protein levels were inhibited by treatment.	[49]
	Tumor xenografts revealed decreased expressions of both VEGF and 12-lipoxygenase proteins in baicalein-treated tumors.	[50]
	Gene expression profiling demonstrated a decrease in both FGFR2 and VEGF following baicalein treatment.	[51]
**Autophagy**	Baicalein induced autophagy and apoptosis in the FRO cells, via regulating the PI3K/Akt and ERK pathways.	[58]
**STAT3**	Baicalein played a role in the inhibition of cell viability and epithelial–mesenchymal transition, and the induction of cell apoptosis, via the enhancement of miR-183 following the JAK2/STAT3 signaling pathway’s inactivation.	[63]
**Wnt/β-catenin**	Baicalein and miR-25 decreased the expressions of Axin2 and β-catenin, whereas expression of GSK-3β increased.	[64]
	Baicalein improved the canonical Wnt/β-catenin pathway through disrupting its translocation from cytoplasm to nucleus.	[65]
**PTEN**	Baicalein inhibited hypoxia-induced Akt phosphorylation through enhancing PTEN accumulation.	[69]
	Baicalein upregulated PTEN expression, downregulated miR-424-3p, and downregulated PI3K and p-Akt.	[70]
**ERK/MAPK**	The combination of baicalin and baicalein treatment caused the activation of ERK and p38 MAPK, and the phosphorylation of p38 MAPK and ERK.	[77]
	Baicalein decreased the phosphorylation of ERK1/2 and MEK1 in a concentration-dependent fashion.	[78]

## 3. Role of Baicalein in Prevention and Treatment of Different Cancers

**Figure 3:**
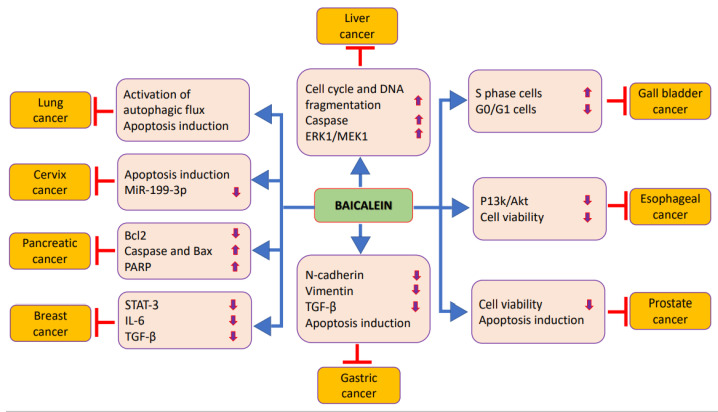
Role of baicalein in different types of cancer.

The important role of baicalein in cancer anticipation and treatment has been established in different cancers, and is related to modifying cell signaling molecules’ activities (Table 2 and Figure 3). The detailed role of baicalein in the prevention and treatment of cancers is explained below. 

### 3.1. Prostate Cancer

Prostate cancer is the most commonly diagnosed cancer in males, being predominantly common in Western countries [79]. Family history, obesity, ethnicity and old age are the risk factors for prostate cancer [80]. The bioactive compounds of herbs play a role in prostate cancer management through modulating various cell signaling pathways. To evaluate the effects of baicalein on prostate cancer proliferation, PC-3 cells were treated with various concentrations of baicalein. The outcome of the study shows that baicalein meaningfully inhibited the proliferation of cancer cells, including PC-3 and DU145 cells, in a time- as well as dose-dependent way. The viability of cancer cells PC-3 and DU145 was reduced to 52.7% and 58.4%, respectively, after baicalein treatment. Furthermore, the IC50 values of baicalein in PC-3 and DU145 cells were between 20 and 40 μmol/L. Besides this, the treatment of PC-3 and DU145 cells with concentrations of 20 and 40 μM baicalein played a role in the induction of apoptosis in a dose-dependent fashion, demonstrating the anti-tumor effect of baicalein [81]. The in vitro effects of baicalein and baicalin on human prostate tumor cells (PC3 and DU-145) and human umbilical vein endothelial cells, and the role of orally administered baicalein in DU-145 cells’ growth after subcutaneous injection into SCID mice, were investigated. The results reveal that baicalin and baicalein showed dose-dependent growth inhibitory effects on umbilical vein endothelial cells and human prostate cancer cells, based on in vitro findings. Moreover, treatment with both compounds meaningfully decreased the length of sprouts formed by the endothelial cell aggregates and their average number in a dose-dependent way. Tumor volume was reduced via the treatment of mice with baicalein as compared to the control [82]. 

### 3.2. Liver Cancer

Baicalein played an important role in the inhibition of the proliferation of malignant liver cells through cell cycle arrest induction at the S and G2/M phases. Besides this, baicalein alters the miRNA expression profiles in liver cancer cells. The regulation of miRNA expression might be a significant cause of the anti-hepatoma effects of baicalein [83]. Baicalein has shown anti-cancer efficiency against human hepatoma cells. The dose–response of baicalein in Hep J2 and Hep G2 cells designates that baicalein reduced the viability by more than 90%. Besides this, baicalein inhibited the cell cycle of Hep G2 cells in the S phase, and baicalein treatment caused damage to the integrity of the cell membrane and decreased mitochondrial transmembrane potential. Moreover, the TUNEL assay results show that baicalein provoked a significant increase in DNA fragmentation in Hep G2 cells [84]. Baicalein, and flavonoids of *Scutellaria baicalensis* or *Scutellaria radix,* showed noteworthy cytotoxicity when used on hepatocellular carcinoma cell lines, whereas minimal cytotoxicity was noticed in normal liver cell line. As compared to 5-fluorouracil, baicalein had a greater effect on liver cancer cells, and treatment with baicalein significantly activated caspase-9 and caspase-3 and decreased mitochondrial transmembrane potential. Besides this, the tumor growth of HCC xenografts in mice was inhibited by baicalein treatment. The induction of apoptosis was noted in baicalein-treated xenograft tumors through the TUNEL assay. Additionally, baicalein treatment significantly reduced the levels of phosphorylation of ERK1/2, MEK1, and Bad based on in vivo and in vitro findings [85]. Another study based on liver cancer reported that NKILA increases the anti-cancer effects of baicalein, based on in vitro and in vivo studies, through the control of nuclear factor-κB (NF-κB) signaling. Moreover, it was implied that the combination of baicalein and NKILA may be beneficial for liver carcinoma [86]. Baicalein had effects on multi-drug-resistant hepatocellular carcinoma cells recognized to be resistant to anti-cancer drugs such as Epirubicin and 5-FU. Flow cytometry analysis demonstrated that treatment with baicalein caused an increase in the intra-cellular concentrations of Epirubicin and Rho123, and in the corresponding group of cells, compared to untreated cells. Furthermore, baicalein induced autophagy and apoptosis, and decreased Bcl-xl and P-gp expression levels [87]. 

### 3.3. Pancreatic Cancer

The anticancer effects of baicalein in human cisplatin-resistant pancreatic carcinoma were investigated. Baicalein induced selective anticancer effects dose-dependently in CAPAN-2 pancreatic cancer cells, with minimal cytotoxicity to normal cells. The expressions of Bcl-2 were decreased, while those of caspase-3 and Bax were increased. Cell migration and invasion analysis discovered that baicalein induced dose-dependent destruction in the migration and invasion of the pancreatic cancer cell line [88]. The effect of baicalein with docetaxel or gemcitabine was examined through the analysis of apoptosis, proliferation, migration and the cell cycle. It was noticed that the proliferation of pancreatic cancer cells was inhibited by baicalein treatment alone. Remarkably, baicalein demonstrates synergism with gemcitabine or docetaxel in the treatment of pancreatic cancer. In accordance with this, baicalein administration suggestively increases the ability of gemcitabine to block cancer cells’ proliferation. Besides this, baicalein in high concentrations was able to arrest pancreatic cancer cells in the S phase, and this finding was confirmed by cell cycle analysis. Additionally, suppression of the migration of pancreatic cancer cells was also observed as result of low concentrations of baicalein in combination with either cancer drug. Markedly, combination treatment showed significant cell apoptosis induction in cancer cells [89]. The proliferation of pancreatic cancer was inhibited by baicalein significantly in a dose-dependent fashion. Besides this, treatment with baicalein induced apoptosis with the activation of caspase-3 and -7 and PARP, and the release of cytochrome c from mitochondria. Baicalein decreased the expression of Mcl-1 seemingly via a transcriptional mechanism. The effect of baicalein on apoptosis was meaningfully reduced by Mcl-1 overexpression, signifying a critical role of Mcl-1 in this process [90]. 

### 3.4. Gastric Cancer

The effect of baicalein, a flavonoid derived from the root of *Scutellaria baicalensis,* on the migration and invasion of cancer cells was examined. It was reported that baicalein inhibited the migration as well as invasion of gastric cancer, and reduced the expression of the metastasis-associated N-cadherin, ZEB1/2 and vimentin [91]. Baicalein inhibits the cell proliferation of gastric cancer cells, and the inhibitory effect was tremendously enhanced when it was combined with cisplatin. Moreover, the combination of baicalein and cisplatin induced apoptosis and autophagy, and suppressed the invasive capability; the effect of this was stronger than that of baicalein or cisplatin alone. Besides this, baicalein modulated the activities of Nrf2/Keap 1 and Akt/mTOR signaling [56]. Baicalein strongly inhibited gastric cancer cell growth. Baicalein strongly prompted arrest at the S phase in the gastric cancer cell line. Besides this, it caused the induction of apoptosis and disturbed the mitochondrial membrane potential in a dose-dependent fashion. The examination of protein expression levels in cancer cells showed enhancement of Bax and a reduction in Bcl-2 in response to baicalein treatment. These results suggest that baicalein plays a vital role in the induction of apoptosis in gastric cancer cells via the mitochondrial pathway [92]. 

### 3.5. Gallbladder Cancer

The effects of baicalein on the growth of the gall bladder cancer cell lines, including SGC996 and GBC-SD, were examined in vitro. Baicalein treatment caused dose- and time-dependent reductions in the cell viability of the used cell lines. Besides this, colony count assessment showed a baicalein-induced decrease in the colony formation ability in a dose-dependent way. A nude mice tumor xenograft was used to confirm the effects of baicalein in vivo. It was observed that tumor size following baicalein treatment was smaller than that in the control group. We measured whether baicalein causes cell cycle progression, and after treatment with baicalein, we saw a noteworthy increase in the number of cells in the S phase and a substantial decrease in the number of cells in the proliferative G0/G1 phase [93].

### 3.6. Bladder Cancer

The rate of cell growth was reduced by the treatment of baicalein in a concentration- and time-dependent fashion. Baicalein in concentrations of 80–120 μmol/L nearly totally blocked cell proliferation. Baicalein treatment caused cancer cell arrest at the G1/S phase, and T24 cells underwent cellular apoptosis after treatment with baicalein. Besides this, the levels of apoptosis were notably higher in cells treated with baicalein than in the control cells [94]. 

Baicalein inhibits the proliferation of cancer cells, and arrests cells in the S phase below 75 μM and in the G1 phase at 100 μM. Besides this, the protein expressions of cyclin D1 and cyclin B1 were decreased by baicalein, and the inhibition of p65NF-*κ*B results in cell growth reduction [95]. The proapoptotic effects of baicalein in human bladder cancer cells were investigated. Baicalein treatment caused concentration-dependent growth inhibition, as well as the induction of apoptosis. Besides this, baicalein regulates apoptotic protein expression, and prominently aggravates the loss of mitochondrial membrane potential. Moreover, the blocking of reactive oxygen species generation decreases the apoptotic activity as well as antiproliferative effects of baicalein, demonstrating that baicalein prompts apoptosis of cancer cells via the reactive oxygen species-dependent activation of caspases [96].

**Table 2:** The role baicalein in different types of cancer.

**Cancers**	**Cell Lines**	**Outcome**	**Refs.**
**Prostate**	PC-3 and DU145	Baicalein inhibits the proliferation of cancer cells in a time- and dose-dependent way.	[81]
		Baicalin and baicalein showed dose-dependent growth inhibitory effects on umbilical vein endothelial cells and human prostate cancer cells.	[82]
**Liver**	Bel-7402	Baicalein inhibits the proliferation of liver cancer cells through cell cycle arrest induction at the S and G2/M phase. Besides this, baicalein alters the miRNA expression profiles in liver cancer cells.	[83]
	Hep J2 and Hep G2	Baicalein inhibited the cell cycle in the S phase and baicalein treatment damaged the integrity of the cell membrane and decreased mitochondrial transmembrane potential.	[84]
	HepG_2_, BEL-7402, SMMC-7721	Baicalein especially inhibits liver tumor growth via MEK-ERK inhibition signaling and by encouraging intrinsic apoptosis.	[85]
**Pancreas**	CAPAN-2	Baicalein induce dose-dependent as well as specific anticancer effects. The antiproliferative effects were seen due to the induction of apoptosis and increased apoptotic cells with increasing the concentration of the used molecule.	[88]
	PANC-1, MIA PaCa-2 and HPAF-II	Baicalein showed synergistic potential with gemcitabine or docetaxel on the treatment of cancer cells.	[89]
	BxPC-3, HPAF-II, Capan-2, AsPc-1, MIA PaCa-2, and Panc-1	Baicalein encourages apoptosis in pancreatic cancer cells via anti-apoptotic Mcl-1 protein downregulation.	[90]
**Gastric**	AGS	Baicalein inhibited the invasion and migration of cancer cells.	[91]
	SGC-7901	Baicalein strongly caused arrest at the S phase. It induced cancer cell apoptosis as well as disturbing the mitochondrial membrane potential in a dose-dependent way.	[92]
**Gall bladder**	GBC-SD and SGC996	Baicalein induced an important inhibitory effect on proliferation and caused apoptosis promotion. Moreover, treatment via baicalein inhibited the metastasis of cancer cells.	[93]
**Urinary bladder**	T24	Baicalein suppressed the inhibition of growth and induction of apoptosis via loss of mitochondrial transmembrane potential and activation of caspase-3 and 9.	[94]
	5637	Baicalein played a role in the regulation of apoptic protein expression via increased Bcl-2 expression and decreased Bax expression.	[96]
**Bile ducts**	HUH28 and TFK1, HUCCT1, QBC939 and MZ-ChA-1	Baicalein showed potential anti-cancer activities through suppressing multiple malignant phenotypes as well as most possibly via inhibiting the activation of the STAT3 and Akt/NF-κB signaling pathways.	[97]
**Colorectal**	HT-29	Baicalein encouraged apoptosis through the activation of Akt in a p53-dependent way in colon cancer cells.	[98]
	HT-29, HCT-116, SW480, and SW620	Inhibition of autophagy increased the induction of apoptotic cell death via baicalein treatment in colon cancer cell lines.	[33]
**Breast**	MDA-MB-231	Baicalein played a role in the inhibition of proliferation, suggesting that baicalein can meaningfully inhibit the proliferation, invasiveness and migration of cancer cells via downregulation of SATB1expression.	[99]
	MCF-7	Baicalein suppress 17beta-estradiol-induced transactivation in cells expressing estrogen receptor alpha.	[100]
	MDA-MB-231 and MCF-7	Co-culturing with M2 macrophages meaningfully enhanced the EMT of both MCF-7 and MDA-MB-231 and cancer cells. Baicalein regulates the polarization of M2 and reduces the secretion of TGF-β1.	[101]
**Cervix**	SiHa and HeLa	Baicalein suspended the cell cycle at the G_0_/G_1_ phase through cyclin D1 downregulation via the signaling pathway of Akt-GSK3β.	[102]
	HeLa, SiHa, ME-180, and Caski	Baicalein downregulated long noncoding RNA (BDLNR) initiating the anti-cancer effects of baicalein.	[103]
	HeLa and SiHa	Baicalin showed anti-cancer effects on cervical cancer cells via STAT3 targeting regulated signaling pathways.	[104]
**Ovarian**	OVCAR-3 and CP-70	Baicalein is more potent in inhibiting cancer cell viability as well as the expression of HIF-1α, vascular endothelial growth factor, cMyc, and NF-κB.	[49]
	SKOV3, and CAOV3	Baicalein inhibits the expression of MMP-2 and the invasion ability of cancer cells, probably via the p38 MAPK-dependent NF-κB signaling pathway.	[105]
**Oesophagus**	EC-109	Baicalein meaningfully inhibits growth and causes the induction of apoptosis in cancer cells.	[106]
**Lung**	A549 and H1299	Baicalein activated AMP-activated protein kinase pathway. The knockdown of AMP-activated protein kinase with lentivirus encoded AMPKα reduced Baicalein-induced mitochondrial fission, autophagy and apoptosis.	[107]
**Bone**	MG-63 and Saos-2	Baicalein inhibits migration, proliferation, as well as invasion, and encourages apoptosis in cancer cells.	[108]
	MG-63 and 143B	Baicalein meaningfully decreases the proliferation of cancer cells in a concentration- and time-dependent fashion. In addition, baicalein induces apoptosis as well as cell cycle arrest and decreases cell motility.	[109]
**Oral**	Cal27	Baicalein enhances autophagy via the promotion of reactive oxygen species signaling pathways in oral cancer.	[110]
	SCC25, CAL27 and HSC3	Baicalein suppresses the growth of oral cancer cells via an Sp1/NF-κB-dependent mechanism.	[111]
	HSC-3	In HSC-3 the decrease in pRb is mediated via baicalein through both the facilitation of cyclin D1 degradation and the activation of aryl hydrocarbon receptor.	[112]
**Thyroid**	8505c ATC	The combination of baicalein and docetaxel meaningfully inhibited proliferation and induced apoptosis. The combination treatment meaningfully inhibited the expressions of VEGF, Bax, TGF-β1, caspase-3, E-cadherin, N-cadherin, and mTOR.	[113]
	MDA-T68	Baicalein induced dose-dependent suppression in the proliferation of thyroid cancer cells. Baicalein induced cell apoptosis in a concentration-dependent fashion.	[57]
**Brain**	U251	Baicalein inhibits glioblastoma cells’ viability and induces apoptosis through inhibiting the activity of NF-kB-p65, suggesting that Baicalein is a potential therapeutic agent for glioblastoma.	[114]
**Skin**	A375 and SK-MEL-2	The suppression of baicalein on melanoma cells through the inhibition of tumor cell glucose uptake and metabolism via affecting the mTOR-HIF-1α signaling pathway.	[115]
	B16F10	Baicalein inhibits melanoma cell migration as well as invasion through decreasing the expression of matrix metalloproteinase and tightening TJ via claudin expression suppression.	[116]
**Blood**	CMK, CMY, Y10, 6133, and 6133 MPL/W515L	Baicalein strongly inhibited proliferation of multiple Akt megakaryoblastic leukemia	[117]
	HL-60	Baicalein-induced apoptosis was noticeably blocked by the broad-spectrum caspase inhibitor.	[118]
	U266, NOP2, AMO1, and ILKM2	Baicalein is a strong inhibitor of protein phosphorylation induced through Interlukin-6, and therefore may be a valuable agent for the treatment of myeloma.	[119]
**Lymphatic**	EL4	Baicalein treatment led to important reductions in the activity of thioredoxin reductase as well as nuclear levels of thioredoxin-1, thus enhancing caspase-3 activity and ASK1 levels.	[120]

### 3.7. Bile Duct Cancer

The anticancer activity and causal mechanisms of baicalein on cholangiocarcinoma were investigated. The results reveal that cell proliferation and viability were inhibited by baicalein treatment in a dose- and time-dependent fashion. Additionally, baicalein plays a significant role in the induction of cell apoptosis and cell cycle arrest by affecting cell cycle-linked proteins and Bcl-2 family proteins expression. More prominently, baicalein suppressed migration as well as invasion through decreasing the aerobic glycolysis and expression of MMP-2/9 proteins. Additionally, baicalein’s role was also suggested by the in vivo results, as it inhibited the tumor growth in subcutaneous xenograft mice models [96].

### 3.8. Colorectal Cancer

Baicalein and wogonin increased the expression of Bax and decreased Bcl-2 expression, in a dose-dependent way, as compared to the control. Additionally, the induction of apoptosis was caused by the inactivation of PI3K/Akt in a dose-dependent fashion. Baicalein administration to mice caused inhibition in the growth of HT-29 xenografts without any toxicity after treatment. The results suggest that baicalein induced apoptosis through Akt activation in a p53-dependent way in the colon cancer cells, and that it might serve as a chemopreventive or therapeutic agent for colon cancer [98]. Human colon cancer cell lines SW480, HCT-116, HT-29, and SW620 were treated with baicalein alone and in combination with chloroquine. Baicalein played role in reducing the cell viability of cancer cells in a dose-dependent manner. The combination treatment of baicalein and chloroquine meaningfully reduced cell viability, as compared with baicalein alone, in cancer cell lines including HCT-116 and HT-29. Cancer cell lines such as HCT-116 treated with both chloroquine as well as baicalein showed increased LC3-II expression levels. The combination of baicalein with chloroquine culminated in the activation of caspase-3-initiated apoptosis [33]. Baicalein induced the upregulation of Decidual protein induced by progesterone (DEPP) as well as Gadd45a, which endorsed the activation of MAPKs with a positive feedback loop between Gadd45a as well as JNK/p38, resulting in a noticeable apoptotic response in human colon cancer cells. These outcome designated that baicalein is a potential antitumor drug for the treatment of colon cancer [121].

### 3.9. Breast Cancer

Breast cancer is well recognized as a genetically as well as clinically heterogeneous illness, with numerous subtypes with distinct molecular characteristics and histopathological patterns, resulting in various responses to therapies and clinical outcomes [122,123]. Baicalein has a proven anti-cancerous role through modulating various cell signaling pathways. Baicalein, a flavone isolated from the roots of Scutelleria baicalensis, powerfully suppressed signal transducer and activator of transcription 3 activity in breast cancer cells. Besides this, baicalein inhibited signal transducer and activator of transcription 3 transcriptional activity and its phosphorylation, and showed anti-proliferative effects in breast cancer cells. Furthermore, baicalein decreased the production of IL-6 as well as the metastatic potential of breast cancer cells, in both in vivo and in vitro conditions [62]. The administration of baicalein suppressed the proliferation of breast cancer MDA-MB-231 cells. Besides this, the administration of baicalein was revealed to meaningfully inhibit the proliferation of MDA-MB-231 cells at various concentrations. The proliferation of cancer cells slowly increased in a time- and dose-dependent manner with administration time and increases in drug concentration [99]. Baicalein played a role in the suppression of 17-beta-estradiol-induced transactivation in cells expressing estrogen receptor alpha, while genistein did not show antagonistic activity and baicalein was a stronger apoptosis-inducing agent than genistein [100]. The potential role of baicalein in the epithelial–mesenchymal transition and macrophage polarization of breast cancer was examined. The in vitro findings suggest that that co-culturing with M2 macrophages meaningfully enhanced the epithelial–mesenchymal transition of both MCF-7 and MDA-MB-231 breast cancer cells. Baicalein controls the polarization of M2 and reduces TGF-β1 secretion. In vivo experiments suggest that the M2 group plus MDA-MB-231, and the tumor growth and metastasis of baicalein + MDA-MB-231 + M2 group, were meaningfully inhibited, with decreased lung metastasis lesions and smaller tumor size [101].

### 3.10. Cervix Cancer

The effect of baicalein on the growth of cervix cancer cells was examined through HeLa and SiHa cells, as these cells were separately treated with different concentrations of baicalein. After treatment, the growth and viability of SiHa cells was slower than that of the control cells. Furthermore, similar results were noted in HeLa cells in the presence of varying concentrations of baicalein. Theses finding suggest that baicalein inhibits the proliferation of HeLa and SiHa cells in vitro, and the inhibitory effect on cell proliferation was dose-dependent [102]. A recent study result showed that baicalein downregulated long noncoding RNA and facilitated the anti-cancer effects of baicalein in cervical cancer through PI3K/Akt pathway activation, and indirectly suggested that baicalein’s downregulation of long noncoding RNA would be potential therapeutic target for enhancing baicalein’s anti-cancer effects [103]. Baicalin treatment decreased cervical cancer cell viability in a concentration-dependent fashion. Treatment with baicalin induced cervical cancer cell apoptosis in a concentration-dependent fashion. Besides this, baicalin treatment inhibited the migration of cervical cancer cells in a concentration-dependent fashion. Baicalin treatment suggestively decreased the wound closure percentage of both SiHa and HeLa cervical cancer cells, and such effects took place in a concentration-dependent way [104]. A recent study reported that cell cycle progression and cell growth were repressed, whereas apoptosis was enhanced, by treatment with baicalein. Besides this, MiR-19a-3p was downregulated in the baicalein-treated cervical cancer cells, and miR-19a-3p overexpression limited baicalein-induced cervical cancer progression inhibition. Baicalein-induced cell cycle arrest, cell growth inhibition, and apoptosis promotion were neutralized through the knockdown of circHIAT1 through targeting miR-19a-3p [44]. Another study found that lncRNA SNHG1 attenuates the tumor-suppressive roles of baicalein in cervical cancer cell viability, migration, apoptosis, and cervical cancer tumor growth [124].

### 3.11. Ovarian Cancer

Baicalein and baicalin inhibited ovarian cancer cell viability in both ovarian cancer cell lines of CP-70 and OVCAR-3. Furthermore, baicalein and baicalin had less inhibitory effects on normal ovarian cells’ viability. Consequently, baicalein is more effective in inhibiting the expressions of VEGF, HIF-1α, cMyc, and NFκB, as well as cancer cell viability, in both ovarian cancer cell lines. It appears that baicalein inhibited cancer cell viability via the inhibition of cancer-promoting genes’ expression, including cMyc, VEGF, HIF-1α, and NFκB [49]. A combined treatment with chloroquine and baicalein seemingly reduced cell viability and increased the cleavage of poly (ADPribose) polymerase (PARP) in both A2780 and HEY ovarian cancer cell lines, suggesting that baicalein protects autophagy in these cells [125]. Baicalein reduced the expression of matrix metalloproteinases-2 in a dose-dependent fashion, and the invasion of ovarian cancer cells was meaningfully inhibited by baicalein. Besides this, baicalein decreased the activation of NF-κB signaling molecules and had an inhibitory effect on p38 activation. Overall, baicalein inhibits the matrix metalloproteinases-2 expression, as well as the invasion ability of ovarian cancer cells, possibly through the p38 MAPK-dependent pathway of NF-κB signaling [105].

### 3.12. Esophageal Cancer

A study was performed to examine whether cultured EC-109 esophageal squamous cell carcinoma cells undergo apoptosis after baicalein treatment, and to examine the underlying mechanisms based on in vitro experimentation. The results show that the treatment of esophageal cancer cells with baicalein evidently decreases the cell viability rate, and colony formation was nearly completely suppressed at a baicalein concentration of 40 μM. Besides this, EC-109 cells showed apoptosis in response to baicalein, and this was found to upregulate apoptotic components of the PI3K/Akt pathway, and downregulate anti-apoptotic components. Finally, baicalein encourages apoptosis in cancer cells via the modulation of the PI3K/Akt pathway, therefore expanding our understanding of the molecular mechanisms of action of baicalein in esophageal carcinoma [106].

### 3.13. Lung Cancer

The association between mitochondrial fission and baicalein-induced apoptosis and autophagy was investigated. It was reported that baicalein induced mitochondrial apoptosis and inhibited cell viability in lung cancer cells. Baicalein induced the loss of mitochondrial membrane potential as well as the apoptosis-inducing factor, and caused the release of cytochrome c from the mitochondria to the cytoplasm. In the meantime, baicalein activated autophagic flux and induced autophagy. In vivo data suggest that baicalein induced apoptosis and autophagy and inhibited tumor growth in a Lewis lung carcinoma xenograft model through the activation of the pathway of AMPK/mitochondrial fission [107].

### 3.14. Bone Cancer

To examine the role of baicalein in osteosarcoma cell proliferation, various concentrations of baicalein were used to treat the cells. The results show that the proliferation of MG-63 and Saos-2 cells was inhibited by baicalein in a concentration- and time-dependent manner. The apoptosis rate of the cancer cell lines, including MG-63 and Saos-2, was investigated following treatment with baicalein. The apoptosis rates of both cancer cells were low in the control group, whereas this effect was meaningfully elevated in the baicalein-treated group, signifying an inductive effect of baicalein on both cancer cell types’ apoptosis. The migration ability of cells was examined after the treatment of cell lines with baicalein (100 µM concentration). It was reported that the migration ability of osteosarcoma cells was noticeably inhibited. Besides this, separately from the migration test, the invasion ability of cancer cells was measured. A similar effect on invasive ability was also noticed as compared to the control group. Overall, baicalein is able to suppress the invasion and migration ability of cancer cells including Saos-2 and MG-63 [108]. The effects of baicalein on osteosarcoma and the probable mode of action were investigated. It was confirmed that baicalein, in a concentration- as well as time-dependent fashion, suppressed the proliferation of cancer cells. Besides this, baicalein may perhaps encourage cell cycle arrest and apoptosis, and caused a cell motility reduction. Furthermore, the level of β-catenin as well as its target genes, such as cyclin D1, c-myc, and survivin, were notably decreased in cancer cells treated with baicalein, while the expression of β-catenin could reverse the anti-metastatic as well as anti-proliferative effects of baicalein. Next, we assessed a 143B xenograft tumor model, wherein treatment with baicalein notably inhibited the growth of tumor by inhibiting the pathway of Wnt/β-catenin. Therefore, these results suggest that the baicalein present in Chinese herbal medicine may be effective for the therapeutic treatment of osteosarcoma [109].

### 3.15. Oral Cancer

The anti-cancer activity and molecular targets of baicalein in oral squamous cell carcinoma were studied in an in vitro medium. The results demonstrate that baicalein induced substantial apoptosis in oral squamous cell carcinoma cells. Besides this, it was reported that baicalein induced an autophagic response in oral squamous cell carcinoma cells. Furthermore, pharmacologically or genetically blocking autophagy improved baicalein-induced apoptosis, demonstrating the cytoprotective role of autophagy in baicalein-treated oral squamous cell carcinoma Cal27 cells. Significantly, it was noticed that baicalein activated reactive oxygen species (ROS) generation in Cal27 cells. Additionally, *N*-acetyl-cysteine, a ROS scavenger, undermined the effects of baicalein on reactive oxygen species-dependent autophagy [110]. The proliferation of oral squamous cell carcinoma cells treated with baicalein was investigated using a CCK-8 assay. Moreover, the effects of baicalein on the apoptosis cell cycle of oral squamous cell carcinoma cells were determined through in vitro and in vivo studies, which reported that baicalein suppresses the proliferation of cancer cell lines. Baicalein arrested the cell cycle at the G_0_/G_1_ phase and caused the induction of apoptosis in such cancer cells. Baicalein inhibited the expression of p65, Sp1, and p50 via downregulating the relative mRNA levels, and decreased the action of NF-κB in cancer cells [111]. Another study demonstrated that cell proliferation was inhibited by baicalein, and that baicalein increased aryl hydrocarbon receptor activity in a dose-dependent fashion, while the cell cycle was arrested at the G1 phase. This finding suggests that the baicalein activation of the aryl hydrocarbon receptor is certainly linked with a reduction in pRb, while it is independent of the reduction in CDK4 and cyclin D1 [112].

### 3.16. Thyroid Cancer

The combination of docetaxel (10 nM) and baicalein (50 or 100 μM) significantly induced apoptosis and inhibited proliferation, as compared with monotherapies. The combination of baicalein and docetaxel treatment increased the expression of caspase-3, Bax, vascular endothelial growth factor, transforming growth factor beta 1, N-cadherin, E-cadherin, and mTOR, but decreased the expression of Bcl-2. The combination of docetaxel and baicalein efficiently induced apoptosis and inhibited metastasis via reducing apoptotic and angiogenic protein expression and stopping the Akt/mTOR and ERK pathways [113].

The proliferation of MDA-T68 thyroid cancer was reduced by baicalein, while the decrease in cell proliferation was significantly less notable for normal thyrocytes. Baicalein caused the induction of cell apoptosis in a concentration-dependent way. The induction of apoptosis was accredited to the increase in apoptotic protein concentration, and the signal was initiated via a change in the Bax/Bcl-2 ratio. Autophagic cell death occurred in cancer cells when treated with baicalein [57]. A recent study demonstrated that baicalein reduced the colony numbers of follicular undifferentiated thyroid cancer cells, as well as their cell viability, in a dose- and time-dependent fashion. Baicalein also arrested the cell cycles and induced cell apoptosis in follicular undifferentiated thyroid cancer cells. Baicalein increased the expressions of Caspase-3 and Caspase-8 and decreased the ratio of Bcl-2/Bax. Besides this, baicalein induced autophagy in cancer cells. It significantly decreased the ratios of p-ERK/ERK and p-Akt/Akt, and increased the expression of Beclin-1, Atg5, p62 and Atg12 [58]. 

### 3.17. Brain Cancer

Baicalein inhibits glioblastoma cell viability and induces apoptosis through the inhibition of NF-kB-p65 activity, suggesting that Baicalein is a potential therapeutic agent for glioblastoma [114]. The anti-tumor role of baicalein in the orthotopic glioma model was investigated. It was reported that that treatment of mice with gliomas by baicalein meaningfully inhibits prolonged survival and intracerebral tumor growth. Furthermore, treatment with baicalein arrested the cell cycle, promoted apoptosis and suppressed the proliferation of cancer cells. Additionally, treatment with baicalein reduced the edema of tumors, decrease the tumor permeability and improved tight junctions. Lastly, treatment with baicalein decreased the expression of Hypoxia-inducible factor 1-alpha, vascular endothelial growth factor, and VEGFR2 in U87 gliomas. Moreover, treatment with baicalein evidently reduced tumor growth and extended the survival of rats [126]. 

### 3.18. Skin Cancer

Baicalein as well as baicalin meaningfully inhibit melanoma cells’ growth and proliferation, suppress tumor cell colony development and migration, and encourage apoptosis as well as senescence in cancer cells. The antitumor effects facilitated by baicalin and baicalein are independent of B-RAF and N-RAS mutation status in cancer cells. Furthermore, it was demonstrated that baicalin and baicalein reduce tumorigenesis and tumor growth in vivo in the melanoma model [115]. The effects of baicalein on cell motility and anti-invasive activity via mouse melanoma B16F10 cells were investigated. The result demonstrates that baicalein pointedly inhibited the cell motility and invasiveness of mouse melanoma B16F10 cells in a concentration-dependent fashion. Besides this, baicalein decreased the activity and expression of matrix metalloproteinase-2 as well as 9; however, the levels of tissue inhibitor of metalloproteinase-1 and -2 were increased. Moreover, treatment with baicalein evidently decreased the expression levels of lipopolysaccharide-induced phosphorylated Akt, as well as the invasive activity of B16F10 cells [116]. 

### 3.19. Leukemia

A study confirmed that baicalein strongly inhibits the proliferation of multiple Akt megakaryoblastic leukemia cells, such as CMY, CMK, Y10, 6133, and 6133 MPL/W515L, due to the apoptosis and arrest of the cell cycle at the G1 phase. Furthermore, baicalein induced the differentiation of 6133 MPL/W515L cells, promoted mouse survival, and decreased disease burden in a mouse model of Akt megakaryoblastic leukemia [117]. The possible apoptosis effects of baicalein on human promyelocytic leukemia cells in vitro were investigated. A substantial difference in cell death in leukemia cells was noticed between baicalein treatment and untreated groups. Additionally, there was an additional significant enhancement in apoptosis induction when cells were treated with baicalein as compared to non-baicalein-treated groups [118]. Baicalein induces the mitochondria-dependent cleavage of caspases-9 and -3, as well as PARP, with concomitant decreases in IAP family proteins, XIAP and survivin. Additionally, baicalein activates the convergence of the intrinsic and extrinsic apoptotic pathways through the death receptor–caspase 8–tBid signaling cascade in CCRF-CEM cells. Moreover, it was reported that the combination of vincristine and baicalein has synergistic therapeutic efficacy [127].

### 3.20. Myeloma

The proliferation and apoptosis rates of myeloma U266 cells exposed to different concentrations of baicalein were noted. Baicalein induced apoptosis and inhibited the growth of myeloma cells in a dose- and time-dependent means. Besides this, baicalein increased the mRNA level of CRBN, and additional studies propose that baicalein downregulates IKZF3 and IKZF1 at the post-transcriptional level [128]. Consistently, it meaningfully reduced the ABCG2 protein expression level. Baicalein also shares similar sites of protein binding and modes with fumitremorgin C. Baicalein has anti-cancer properties; for example, it causes anti-proliferation as well as drug efflux inhibition in side population cells, which suggests its potential capacity for directing cancer stem cells in multiple myeloma [129]. Baicalein causes the apoptosis of myeloma cell lines through decreasing the expression of IL-6 and inhibiting the phosphorylation of XIAP and IκB-α genes. This leads to the activation of caspase-9 and -3, and changes in mitochondrial membrane potential. Baicalein is a potent inhibitor of protein phosphorylation induced by IL-6, and thus may be a useful agent for the treatment of multiple myeloma [119].

### 3.21. Lymphoma

The effect of baicalein on murine T cell lymphoma EL4 cells and the causal mechanism of action were examined. Baicalein induced cell death dose-dependently in cancer cells in vitro, and suggestively decreased the frequency of cancer stem cells. Baicalein treatment led to a substantial decrease in the activity of thioredoxin reductase, as well as nuclear levels of thioredoxin-1, thus increasing ASK1 levels and caspase-3 activity. Additionally, baicalein treatment meaningfully decreased the intra-peritoneal tumor burden of EL4 cells in C57BL/6 mice [120]. 

## 4. Bioavailability and Strategies to Improve the Baicalein Delivery

Baicalein’s health promoting effects have been demonstrated through its antioxidant, anti-inflammatory, anti-microbial and anti-cancerous activities. However, in spite of its various health-promoting effects, the powers of baicalein in curing disease cure are restricted by its low aqueous solubility, rapid metabolism and poor oral absorption. After the oral administration of three doses of baicalein, plasma concentration–time curves (multi-peaks) were detected, and the non-linear pharmacokinetics of baicalein were assessed at doses of 50–500 mg/kg. The absolute bioavailability was calculated, and an intravenous pharmacokinetic study was also carried out after intravenous administration of 10 mg/kg baicalein. The complete bioavailability of baicalein in different doses ranged from 13.1 to 23.0% [130]. 

As per Phase I, a randomized, double-blind, single-dose trial of baicalein (100–2800 mg) was performed on seventy-two healthy subjects. Samples of blood, urine and feces were obtained at consistent breaks up to 48 h after administration of the tested drug. Baicalein was then examined using liquid chromatography–tandem mass spectrometry. The total urinary clearance of baicalein was observed to be below 1%. Almost 27% of the baicalein was removed unchanged via the feces. Single oral doses of 100–2800 mg of baicalein were well tolerated and safe for healthy subjects [131]. Thus, numerous findings suggest this substance can overcome the problems of low aqueous solubility, fast metabolism and poor oral absorption. Several studies have found that nanotechnology-related approaches can be utilized in delivery to improve the bioavailability of baicalein (Table 3).

An important study prepared baicalein–theophylline (BE-TH) co-crystal nanocrystals to attain simultaneous improvements in the dissolution as well as oral bioavailability of baicalein. The results demonstrate that compared with a coarse powder of baicalein, baicalein–theophylline (BE-TH) co-crystals significantly improve the solubility of baicalein. The dissolution test exhibited that the baicalein–theophylline co-crystals showed higher rates of dissolution than the baicalein coarse powder in hydrochloric acid as well as phosphate buffer. Furthermore, after the oral administration of the co-crystals, it was reported that the co-crystals showed a 5.86-times higher area under the curve in rats [132]. The potential of oral and pulmonary nanocrystals to improve the bioavailability of baicalein was examined. The baicalein nanocrystal was made via anti-solvent recrystallization. The pulmonary baicalein nanocrystal showed rapid as well as wide absorption, and had almost equal pharmacokinetic parameters to intravenous the baicalein injection [133]. 

A self-microemulsifying drug delivery system (SMEDDS) for the oral bioavailability enhancement of a poorly water-soluble baicalein was investigated. The drug release rate of the self-microemulsifying drug delivery system was notably higher than that of the baicalein suspension. A comparison of the pharmacokinetics between the baicalein-loaded self-microemulsifying drug delivery system and the baicalein suspension was also performed. The in vivo results show that the absorption of baicalein from the self-microemulsifying drug delivery system caused an increase in the relative bioavailability, as compared with that of the baicalein suspension [134]. The interaction of 2-hydroxypropyl-beta-cyclodextrin with baicalein and 5,6,7-trihydroxy flavone in solution and solid-state was examined. Through complexation with 2-hydroxypropyl-beta-cyclodextrin, the solubility of baicalein in neutral aqueous solution was enhanced. Baicalein/2-hydroxypropyl-beta-cyclodextrin solid powders were amorphous, and showed a meaningfully better dissolution rate in comparison with free baicalein. The in vivo results show that the Baicalein/2-hydroxypropyl-beta-cyclodextrin co-lyophilized product exhibits similar pharmacokinetics to free baicalein after intravenous administration. The Baicalein/2-hydroxypropyl-beta-cyclodextrin co-lyophilized product displays earlier tmax and higher Cmax of BG than free baicalein after oral dosing [135]. 

Long-circulating nanoliposomes (LCNs) consisting of baicalein were made via the diethyl ether injection method. A drug storage stability study showed that Baicalein-loaded long-circulating nanoliposomes were highly stable. Moreover, long-circulating nanoliposomes-encapsulated baicalein achieves greater baicalein oral bioavailability, with a value 4.52 times that of free baicalein [136]. The effects of the preparation method on co-crystallization between baicalein and nicotinamide (NCT), their intermolecular interaction, and whether BE-NCT co-crystals can achieve simultaneous enhancements in the solubility, dissolution, and oral bioavailability of baicalein, were investigated. The results reveal that, compared with crystalline baicalein, BE-NCT co-crystals showed meaningfully better solubility and dissolution of baicalein values. Furthermore, the co-crystals exhibited a 2.49-fold higher peak plasma concentration (Cmax), as well as a 2.80-fold higher area under the curve (AUC), in rats [137]. Baicalein was formulated into liposomes of numerous sizes to enhance its solubility and stability. The liposomal baicalein inhibited leukemia cell growth efficiently, demonstrating that the liposome may be a potential vehicle to deliver baicalein for the treatment of leukemia. We report cell cycle arrest in the sub-G1 phase, representative of cell apoptosis, following the 20 µM liposomal baicalein or free baicalein treatment of cancer cells [138]. Baicalein-loaded nanoliposomes were prepared to improve the bioavailability, and it was reported that baicalein-loaded nanoliposomes can achieve improved antitumor effects compared to free baicalein. Besides this, the results establish that baicalein-loaded nanoliposomes showed better antitumor effects, with a higher inhibition rate percentage, than free baicalein using a U14 cervical tumor-bearing mice model [139].

**Table 3:** Strategies to improve baicalein delivery.

Nanoformulation	Outcome	Refs.
Baicalein–theophylline (BE-TH) co-crystals	Baicalein–theophylline (BE-TH) co-crystals significantly improved the solubility of baicalein. Co-crystals confirmed higher rates of dissolution than baicalein in hydrochloric acid as well as phosphate buffer.	[132]
Baicalein nanocrystal	The pulmonary baicalein nanocrystal showed fast as well as extensive absorption, and had nearly the same pharmacokinetic parameters as intravenous baicalein injection.	[133]
Baicalein-loaded aelf-microemulsifying drug delivery	The absorption of baicalein from the self-microemulsifying drug delivery system resulted in an increase in the relative bioavailability (about 200.7%) as compared with that of the baicalein suspension.	[134]
Baiclein-2-hydroxypropyl-beta-cyclodextrin (HP-beta-CD)	After intravenous administration, the Ba/HP-beta-CD co-lyophilized product displays similar pharmacokinetics to free baicalein.	[135]
Baicalein-loaded long-circulating nanoliposomes (LCNs)	Long circulating nanoliposome-encapsulated baicalein yields better oral bioavailability.	[136]
Co-crystallization between baicalein and nicotinamide (NCT)	BE-NCT co-crystals enabled meaningfully better solubility and dissolution of baicalein	[137]
Liposomal baicalein	Liposomal baicalein inhibited leukemia cell growth, demonstrating that the liposome may be a potential vehicle to deliver baicalein for the treatment of leukemia.	[138]
Baicalein-loaded nanoliposomes (BAI-LP)	BAI-LP showed good antitumor effects, with a higher inhibition rate percentage than free baicalein.	[139]

## 5. Synergistic Effect of Baicalein in Combination with Anti-Cancerous Drugs against Cancer Cells

**Figure 4:**
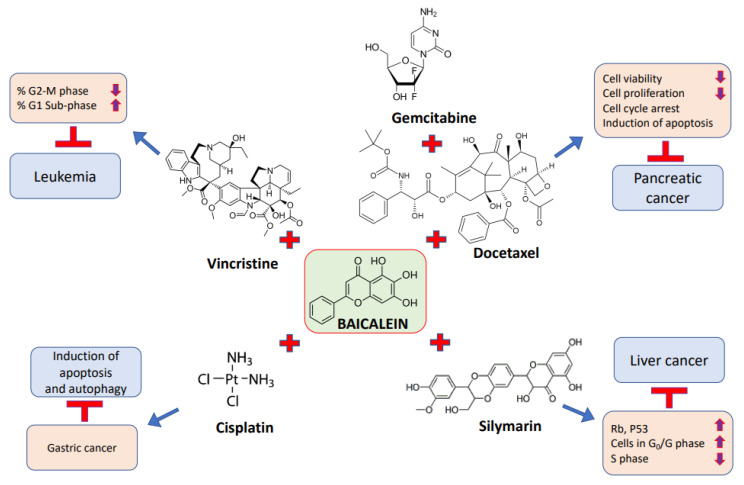
Synergistic effect of baicalein in cancer.

The combination of baicalein with other compounds or anti-cancer drugs is more effective than using the compound alone at equal concentrations (Table 4 and Figure 4). Baicalein, a type of flavonoid, was examined for its role in pancreatic cancer. The treatment with baicalein only meaningfully inhibits the proliferation of pancreatic cancer cells. Notably, when it was combined with gemcitabine or docetaxel, the synergism enhanced its usefulness for the treatment of pancreatic cancer cells [89]. 

The effects of silymarin, baicalein, and their combination on human liver-derived cell lines and hepatocellular carcinoma were investigated. It was reported that the growth of liver cancer cells was significantly inhibited by 100 µg/mL silymarin and 6.75 µg/mL baicalein alone. When it was used in combination with silymarin on liver cancer cells, an additive effect and a synergistic effect were seen. The viability was significantly decreased when given with silymarin as compared to baicalein alone. The results suggest that the combination of baicalein and silymarin destroys tumor cells efficiently, and has minimal deleterious effects on nearby normal cells [140]. The molecular apoptotic mechanisms of baicalein in CCRF-CEM leukemic cells were assessed, and we sought to understand the combined therapeutic efficacy of baicalein with numerous widely used chemotherapeutic drugs in CCRF-CEM cells. The findings reveal that baicalein induces PARP with concomitant decreases in IAP family proteins, XIAP and survivin, and the mitochondria-dependent cleavage of caspases-9 and -3. In addition, the results show that baicalein activates a convergence of the extrinsic and intrinsic apoptotic pathways via the death receptor–caspase 8–tBid signaling cascade. Furthermore, a synergistic therapeutic efficacy was noted in the combination of baicalein with vincristine [127]. 

The synergistic anti-cancer effect of the combination of baicalein and cisplatin on gastric cancer was investigated. The results show that baicalein could inhibit the cell proliferation of HGC-27, MGC-803, SGC-7901/DDP and SGC-7901, and the inhibitory effect was enormously increased when combined with cisplatin. Furthermore, combination of baicalein and cisplatin suppressed the invasive capability, as well as inducing apoptosis and autophagy [56]. The role of baicalein in hypoxia-caused 5-fluorouracil (5-FU) resistance in gastric cancer was investigated. The results show that baicalein enhances the sensitivity of gastric cancer cells to 5-FU treatment under hypoxia. Additionally, baicalein inhibited hypoxia-induced Akt phosphorylation via enhancing PTEN accumulation, thereby reducing hypoxia-inducible factor-1α expression in cancer cells [69].

**Table 4:** Synergistic effect of baicalein in combination with anti-cancerous drugs/compounds.

Cancer	Anti-Cancer Drug/Compound	Effects	Refs.
Pancreatic cancer	Gemcitabine/docetaxel	Baicalein (low concentration) in combination with either gemcitabine or docetaxel achieved the powerful suppression of the migration of cancer cells.	[89]
Liver cancer	Silymarin	Baicalein in combination with silymarin caused an additive effect and a synergistic effect.	[140]
Leukemia	Lincristine	Synergistic therapeutic efficacy was noted in the combination of baicalein with vincristine.	[127]
Gastric cancer	Cisplatin	The combination of baicalein and cisplatin suppressed invasive capability and induced apoptosis and autophagy.	[56]

## 6. Conclusions

Cancer is one of the major causes of death worldwide, and its incidence is increasing. The current modes of treatment, such as surgery, chemotherapy and radiotherapy, may be effective, but such treatments cause adverse effects. Some of the common effects include hair loss, anemia, vomiting, gastrointestinal problems, fatigue, and mouth, gum and throat soars. In addition, radiotherapy- and chemotherapy-based treatments also cause adverse effects on normal cells, and disturb normal physiology. Keeping all these complication in mind, health management researchers are looking for effective treatment modules with the fewest side effects. In this regard, natural products and their bioactive compounds have proven to be effective in disease management, including for cancer. Herbs and their bioactive ingredients play a health-promoting role through the modulation of various biological activities. Among such herbs and bioactive compounds, baicalein shows substantial health-promoting effects, with anti-cancer potential. Baicalein has been revealed to activate multiple mechanisms to manage cancer development and progression by modulating cell signaling pathways, including those related to inflammation, cell cycle, angiogenesis, apoptosis, autophagy, and tumor necrosis factor. This wide-ranging study based on in vivo and in vitro tests has described the role of baicalein in various types of cancer management. The synergistic effects of baicalein in combination with some anticancer drugs have proven very effective therapeutic treatment. Baicalein in combination with silymarin, gemcitabine/docetaxel, and cisplatin has shown effects on cancer inhibition through the effective suppression of the migration of cancer cells, reduced cell viability, the induction of apoptosis as well as the modulation of other biological activities. However, further research is required to investigate the role of baicalein use in cancer prevention and treatment, and in addition, its detailed mechanism of action in cancer management needs to be determined.

## Data Availability

Not applicable.
